# Renal Mg handling, FXYD2 and the central role of the Na,K‐ATPase

**DOI:** 10.14814/phy2.13843

**Published:** 2018-09-02

**Authors:** Haim Mayan, Zvi Farfel, Steven J. D. Karlish

**Affiliations:** ^1^ Department of Medicine E Sheba Medical Center Ramat Gan Israel; ^2^ Laboratory of Biochemical Pharmacology Sheba Medical Center Ramat Gan Israel; ^3^ Sackler School of Medicine Tel Aviv University Tel Aviv Israel; ^4^ Department of Biomolecular Sciences Weizmann Institute of Science Rehovoth Israel

**Keywords:** FXYD2, Na, K‐ATPase, renal Mg handling, tubulopathies

## Abstract

This article examines the central role of Na,K‐ATPase (*α*1*β*1FXYD2) in renal Mg handling, especially in distal convoluted tubule (DCT), the segment responsible for final regulation of Mg balance. By considering effects of Na,K‐ATPase on intracellular Na and K concentrations, and driving forces for Mg transport, we propose a consistent rationale explaining basal Mg reabsorption in DCT and altered Mg reabsorption in some human diseases. FXYD2 (*γ* subunit) is a regulatory subunit that adapts functional properties of Na,K‐ATPase to cellular requirements. Mutations in FXYD2 (G41R), and transcription factors (HNF‐1B and PCBD1) that affect FXYD2 expression are associated with hypomagnesemia with hypermagnesuria. These mutations result in impaired interactions of FXYD2 with Na,K‐ATPase. Renal Mg wasting implies that Na,K‐ATPase is inhibited, but in vitro studies show that FXYD2 itself inhibits Na,K‐ATPase activity, raising K_0.5_Na. However, FXYD2 also stabilizes the protein by amplifying specific interactions with phosphatidylserine and cholesterol within the membrane. Renal Mg wasting associated with impaired Na,K‐ATPase/FXYD2 interactions is explained simply by destabilization and inactivation of Na,K‐ATPase. We consider also the role of the Na,K‐ATPase in Mg (and Ca) handling in Gitelman syndrome and Familial hyperkalemia and hypertension (FHHt). Renal Mg handling serves as a convenient marker for Na,K‐ATPase activity in DCT.

Regulation of plasma Mg concentration (0.7–1 mmol/L) is crucial for normal physiological function. Hypomagnesemia (Mg < 0.7 mmol/L) is the most frequent manifestation associated with Mg imbalance and must be treated with dietary Mg supplements (de Baaij et al. 2015a). Regulation of plasma Mg reflects the balance of intestinal absorption, storage, in bone, muscle and soft tissues, and renal excretion. In kidneys, 90–95% of filtered Mg is reabsorbed, 10–25% in proximal tubules (PCT), about 70% in the thick ascending limb (TALH) and about 10% in distal convoluted tubule (DCT). Reabsorption of Mg in DCT is crucial to the final balance of Mg, because changes in Mg reabsorption in PCT and TALH can be compensated for in the DCT, but not more distally in the collecting duct. Mg reabsorption in PCT and TALH is paracellular, while that in DCT is an active transcellular process (de Baaij et al. 2015a).

Comprehensive reviews of renal Mg handling in health and disease have been published recently (de Baaij et al. 2015a; McCormick and Ellison 2015). We focus here on the role played by the Na,K‐ATPase in renal Mg handling, and inherited diseases of Mg balance, with reference primarily to the regulatory FXYD2 subunit. Although the mechanisms of Mg transport in kidneys are quite well understood, the role of the Na,K‐ATPase has been less clearly defined. Na,K‐ATPase hydrolyses ATP and actively extrudes 3Na ions in exchange for 2K ions per cycle. In the kidney, over 50% of the cellular ATP is utilized by Na,K‐ATPase and even small changes in rates of active Na and K transport can have significant effects on cellular Na and K concentrations, and driving forces for transport of other ions such as Mg or Ca. Na,K‐ATPase activity varies along the renal tubule and, indeed, the highest Na,K‐ATPase activity is found in DCT followed by medullary TALH and PCT (Katz et al. 1979).

Na,K‐ATPase consists of *α* and *β* subunits together with a regulatory FXYD subunit in 1:1:1 molecular complex (*αβ*FXYD) (Kaplan 2002; Jorgensen et al. 2003). *α* subunits consist of about 1000 residues with ten transmembrane segments and contain the functional sites for Na, K and ATP binding. *β* subunits are highly glycosylated single transmembrane segment proteins of about 300 residues, essential for stabilization of the *αβ* complex and normal trafficking and expression in the membrane. There are four isoforms of the *α* (*α*1‐4) and three isoforms of the *β* subunit (*β*1‐3) (Blanco and Mercer 1998). *α*1 is expressed in virtually all cells, and in the kidney the *α* subunit is expressed almost exclusively as *α*1. Other *α* isoforms are expressed in a tissue‐specific fashion. Essentially all *α*1 subunits are complexed with *β*1 (*α*1*β*1).

FXYD proteins are single transmembrane regulatory subunits of 60–70 residues (except FXYD5 with about 140 residues) (Sweadner and Rael 2000). In mammals, there are seven members (FXYD1‐7), expressed in a tissue‐specific fashion. FXYD proteins are not essential subunits but modulate the activity of Na,K‐ATPase (Sweadner and Rael 2000; Garty and Karlish 2006; Geering 2006). In kidney, Na,K‐ATPase (*α*1*β*1) is complexed with FXYD2 (gamma subunit) in all segments of the nephron except for collecting duct principal cells in which it is complexed with FXYD4. FXYD2 is found as two splice variants, FXYD2a and FXYD2b that differ in their extracellular N‐terminal sequences and are expressed along the nephron in a partially overlapping pattern (Kuster et al. 2000; Pu et al. 2001; Arystarkhova et al. 2002). FXYD2a and FXYD2b have similar functional effects but there may be differential effects on cell growth (Wetzel et al. 2004; Capasso et al. 2006).

## Mg Transport in DCT

**Figure 1:**
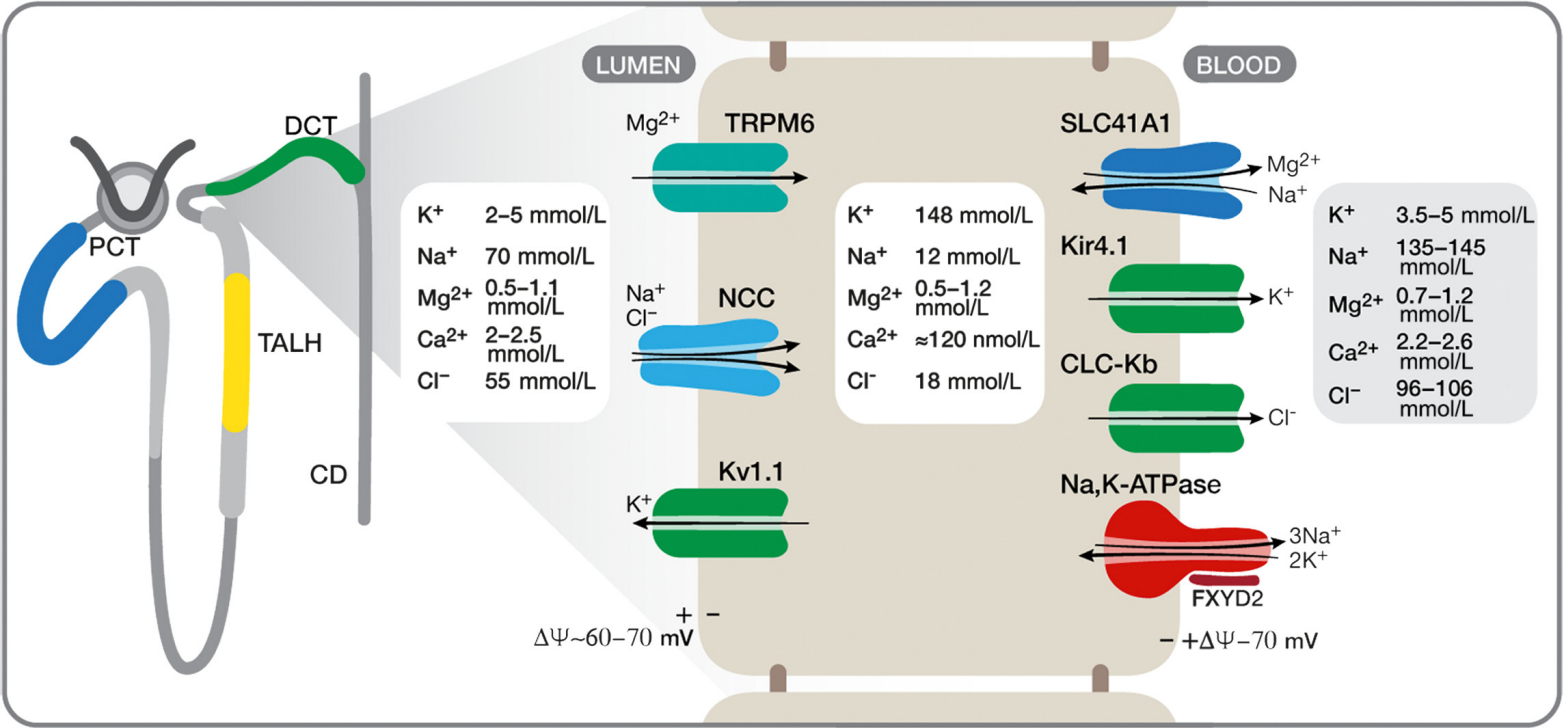
Mg reabsorption in early distal convoluted tubule. The model is adapted from that in Schaffers et al. (2018). Concentrations of ions are taken from references McCormick and Ellison (2015) and Weinstein (2005).

There is now rather extensive knowledge of the mechanism of active Mg uptake and Mg transporters in DCT (Schaffers et al. 2018). Figure 1 shows a simplified schematic cellular model of Mg transport in DCT adapted from (Schaffers et al. 2018). At the luminal surface, the relevant transporters are NCC (SLC12A3), the sodium chloride co‐transporter, TRPM6 (and TRPM7), the Mg channels and Kv1.1, a K channel. At the basolateral surface, the relevant transporters are the Na,K‐ATPase (*α*1*β*1FXYD2), SLC41A1 a sodium/magnesium exchanger, Kir4.1 a K channel, and ClC‐Kb a chloride channel.

Changes in Na/K‐ATPase activity in DCT can be expected to affect intracellular Na and K concentrations and the driving force for transepithelial Mg transport. Although predictions for different conditions are rather clear‐cut, they are necessarily qualitative due to lack of direct experimental evidence on intracellular Na and K concentrations, especially in conditions of abnormal Mg handling. Expected resting concentrations of luminal, intracellular and plasma Na, K, Mg, Ca and Cl concentrations in the early segment of DCT are shown in Figure 1. In DCT, the resting intracellular Mg concentration is 0.5–1.2 mmol/L while the tubular and plasma Mg concentrations are similar, in the mmol/L range (McCormick and Ellison 2015). In the absence of an inwardly directed Mg concentration gradient, the principal driving force for luminal Mg uptake is the membrane potential (Δ*ψ* ≈60–70 mV positive outside). This is primarily a K diffusion potential dependent on the outwardly directed K gradient (intracellular c.148 mmol/L, luminal 5 mmol/L) via the Kv1.1 channel. Mg efflux at the basolateral surface is thought to be mediated by the sodium/Mg exchanger, SLC41A1, which is dependent on the inward Na electrochemical gradient (intracellular Na 10–15 mmol/L, plasma Na, 140 mmol/L, Δ*ψ* ≈−70 mV positive outside). Net changes of intracellular Na and K concentrations should then affect the driving forces for both luminal Mg entry and basolateral Mg exit.

Inhibition of Na,K‐ATPase activity will reduce active basolateral Na efflux and K influx. This should lead to raised intracellular Na, thus reducing the inward Na gradient‐driving basolateral Mg exit, and also lower intracellular K concentrations, reducing the outward K gradient and membrane potential driving luminal Mg entry. Changes in intracellular Na concentration caused by changes in luminal Na entry via NCC will also affect Na,K‐ATPase activity, which is normally limited by intracellular Na (10–15 mmol/L) at about 20% of the Vmax. Reduced Na entry on NCC should lead to both a reduced intracellular Na concentration and reduced Na,K‐ATPase activity with reduced active K uptake. On the contrary, increased Na entry on NCC should increase intracellular Na and also the Na,K‐ATPase activity and increase active K uptake.

For comparison with Mg transport, Ca uptake in DCT is mediated by a luminal Ca channel TRPV5 and basolateral Na/Ca exchanger, NCX1, and plasma membrane Ca‐ATPase (PMCA) (McCormick and Ellison 2015). Note, however, that the driving forces for transepithelial Ca and Mg transport differ. With resting intracellular Ca c. 120 nM and luminal Ca in the mmol/L range (McCormick and Ellison 2015), there is a large inward concentration gradient driving luminal Ca entry. Intracellular Ca concentration is strongly dependent on basolateral sodium/calcium exchange, via NCX1. Thus, inhibition of Na,K‐ATPase activity will reduce the inward basolateral Na gradient and raise intracellular Ca, so reducing the driving force for luminal Ca entry.

## Inherited Mg Wasting Diseases Involving FXYD2

Autosomal dominant renal hypomagnesemia associated with hypocalciuria was described in a large Dutch family and later attributed to a heterozygous mutation in FXYD2, namely G41R within the transmembrane segment (Meij et al. 2000). More recently two additional families with isolated dominant hypomagnesemia and the G41R mutation were identified (de Baaij et al. 2015b). Expression studies showed that the mutant G41R protein was incorrectly routed and accumulated in intracellular organelles (Meij et al. 2000). Renal Mg wasting was suggested to involve a reduction in Na,K‐ATPase activity in DCT cells, and reduced Mg uptake, although no specific mechanism was available. Subsequent experiments in cultured cells confirmed that the G41R mutation prevents trafficking of FXYD2 but not the *α*1*β*1 complex to the cell membrane (Pu et al. 2002).

Independent observations that connect hypomagnesemia and renal Mg wasting with disruption of the FXYD2–*αβ* interaction include identification of mutations in the hepatocyte nuclear factor (HNF‐1B), a transcription factor, expressed in various organs, including kidneys, and associated with abnormal renal development (Adalat et al. 2009). Highly conserved HNF‐1B recognition sites were detected in the FXYD2 gene, showing that HNF‐1B regulates transcription of FXYD2 and tubular handling of Mg. More recently, it was shown that HNF‐1B activates FXYD2a (but not FXYD2b) expression while mutations associated with hypomagnesemia do not activate (Ferre et al. 2011). Since both FXYD2a and FXYD2b were identified in basolateral membranes of DCT cells, it was suggested that the HNF‐1B mutations affect FXYD2a abundance and transcellular Mg transport. Finally, mutations in another gene PCBD1 also cause hypomagnesemia and renal Mg wasting. PCBD1 binds to HNF‐1B and co‐stimulates the FXYD2 promoter but several PCBD1 mutations cause reduced FXYD2 promoter activity and FXYD2 transcription (Ferre et al. 2014).

Overall, it is clear that hypomagnesemia with renal Mg wasting (and hypocalciuria) observed in these genetic diseases is associated with disruption of the normal Na,K‐ATPase–FXYD2 interaction. Can these clinical observations be explained by known functional effects of FXYD2?

## Functional Effects of FXYD2

In vitro effects of FXYD proteins have been studied extensively using transfected cells (Xenopus oocytes or cultured mammalian cells) expressing *αβ* subunits without or with the FXYD protein (Garty and Karlish 2006; Geering 2006). Compared to the control, FXYD2 induces a moderately raised K_0.5_Na and reduced KmATP (1.5‐2‐fold) for Na,K‐ATPase activity (Arystarkhova et al. 1999; Beguin et al. 2001; Pu et al. 2002; Blostein et al. 2003). A slightly reduced or increased K_0.5_K induced by FXYD2 has also been observed in different cells (Beguin et al. 2001).^1^ Observation of more than one kinetic effect in vitro could imply that, at the molecular level, there are several interactions of FXYD2 with the *αβ* subunits. However, at the physiological level, the most significant effect of FXYD2 is likely to be the raised K_0.5_Na because, at normal intracellular Na and K concentrations (Na, 10–15 mmol/L; K, c.120 mmol/L) Na,K‐ATPase activity is far below Vmax. In whole cells, active Na extrusion should increase at raised intracellular Na concentration and decrease at lowered intracellular Na concentration.^2^ For example, in nephron segments with a high luminal Na concentration (PCT or TALH, 140 mmol/L) and a high rate of Na entry, a relatively elevated K_0.5_Na is required for an optimal response to changes in intracellular Na concentration. By contrast, principal cells of collecting duct that express FXYD4 are built to fully reabsorb Na at low luminal Na concentration (≈30 mmol/L). Optimal Na reabsorption is served best by a lower K_0.5_Na and a rate of active Na efflux closer to the Vmax condition. Indeed, FXYD4 significantly reduces K_0.5_Na compared to the control and even more compared to FXYD2 (Beguin et al. 2001; Garty et al. 2002).

Another approach to functional analysis is the use of FXYD2 knock‐out mice. In an initial publication, no effects on urinary Na and K secretion were found in either heterozygous FXYD2^+/−^ or homozygous FXYD2^−/−^ animals (Jones et al. 2005). Urinary Mg was also measured and although pronounced hypermagnesuria was not observed, the published data appear to show a moderate but significantly increased urinary Mg secretion in the FXYD2^−/−^ mice (see Table 2 in (Jones et al. 2005) mg/mg creatinine mean ±  SEM: FXYD2^+/+^ vs. FXYD2^−/−^ :0.86 ± 0.09 (*n* = 15) vs. 1.27 ± 0.15 (*n* = 15) Δ = 0.41 ± 0.175, two‐tailed *t*‐test *P* = 0.0264). Other observations include a somewhat reduced K_0.5_Na for activation of partially purified Na,K‐ATPase, prepared from FXYD2^−/−^ renal cortical microsomes, with little or no change in *α* and *β* subunit expression. Strikingly also, the renal Na,K‐ATPase from FXYD2^−/−^ mice was thermally unstable compared to the wild‐type Na,K‐ATPase (Jones et al. 2005). More recently (Arystarkhova et al. 2014), *α*1*β*1 expression was reported to be unchanged in FXYD2^−/−^ renal cortex, including DCT, although this was assessed by immunofluorescence and small changes, which may have disproportionately large physiological effects, could be hard to detect. In addition, specific Na,K‐ATPase activity of renal cortical microsomes of FXYD2^−/−^ mice showed a rough 25% increase compared to the wild type in *V*max conditions. Nevertheless, absolute Na,K‐ATPase activity was low and could have been partly underestimated because the membranes were not treated with low concentrations of detergent used to unmask closed vesicles. Immunofluorescence and Western blots showed increased abundance of NCC in DCT and strongly increased phosphorylation of both NCC and the sodium, potassium, two chloride co‐transporter (NKCC2) in TALH, which is assumed to reflect increased activity of both co‐transporters. Paradoxically, Na retention and hypertension were not observed. It was suggested that “Activation of NCC and NKCC2 may reflect an intracellular linkage to elevated Na,K‐ATPase activity or a compensatory response to a Na loss more proximal to TALH and DCT” (Arystarkhova et al. 2014). As discussed below, the latter possibility is more likely to be correct.

The inhibitory kinetic effects of FXYD2 in expression experiments, and apparently reduced K_0.5_Na and raised Vmax in the FXYD2^−/−^ knock out kidney membranes, appear mutually self‐consistent. However, based on the assumption that FXYD2 is an inhibitor, a serious problem arises in any attempt to explain hypermagnesuria in human FXYD2 G41R and HNF‐1B mutant diseases. If, disruption of the renal Na,K‐ATPase–FXYD2 interaction causes the predicted increase in Na,K‐ATPase activity and basolateral active K uptake, intracellular K concentration should rise and so increase rather than decrease the driving force for Mg entry in DCT.

A key recent observation suggests a resolution of this paradox. Administration of digoxin to human patients to control rapid atrial fibrillation is associated with increased fractional excretion of Mg in the urine, Fex Mg, with borderline increased Fex Ca but unchanged Fex Na and K (Abu‐Amer et al. 2018). The effect is acute, occurring maximally after one hour and there is a striking correlation between Fex Mg and serum digoxin concentrations, which is in the range 0–5 nmol/L (*r* = 0.68, *P* < 0.0001). These observations leave little doubt that the in vivo action of digoxin is caused by its classical effect to inhibit the Na,K‐ATPase. Indeed, digoxin should inhibit Na,K‐ATPase in all segments of the kidney (PCT, TALH and DCT) raising the intracellular Na and lowering the intracellular K concentrations. The bulk of Mg reabsorption occurs via paracellular pathways in PCT (10–25%) and TALH (70%) driven by the transepithelial electrical potential, and should be reduced by inhibition of the Na,K‐ATPase. In principle, increased delivery of Mg to the DCT could be compensated, at least partially, by increased reabsorption in the DCT, but this will not be possible if Na,K‐ATPase is inhibited also in the DCT. Since inhibition of active Na and K pumping by digoxin in DCT explains hypermagnesuria, could a similar mechanism explain the Mg wasting associated with disrupted *αβ*–FXYD2 interactions?

**Figure 2:**
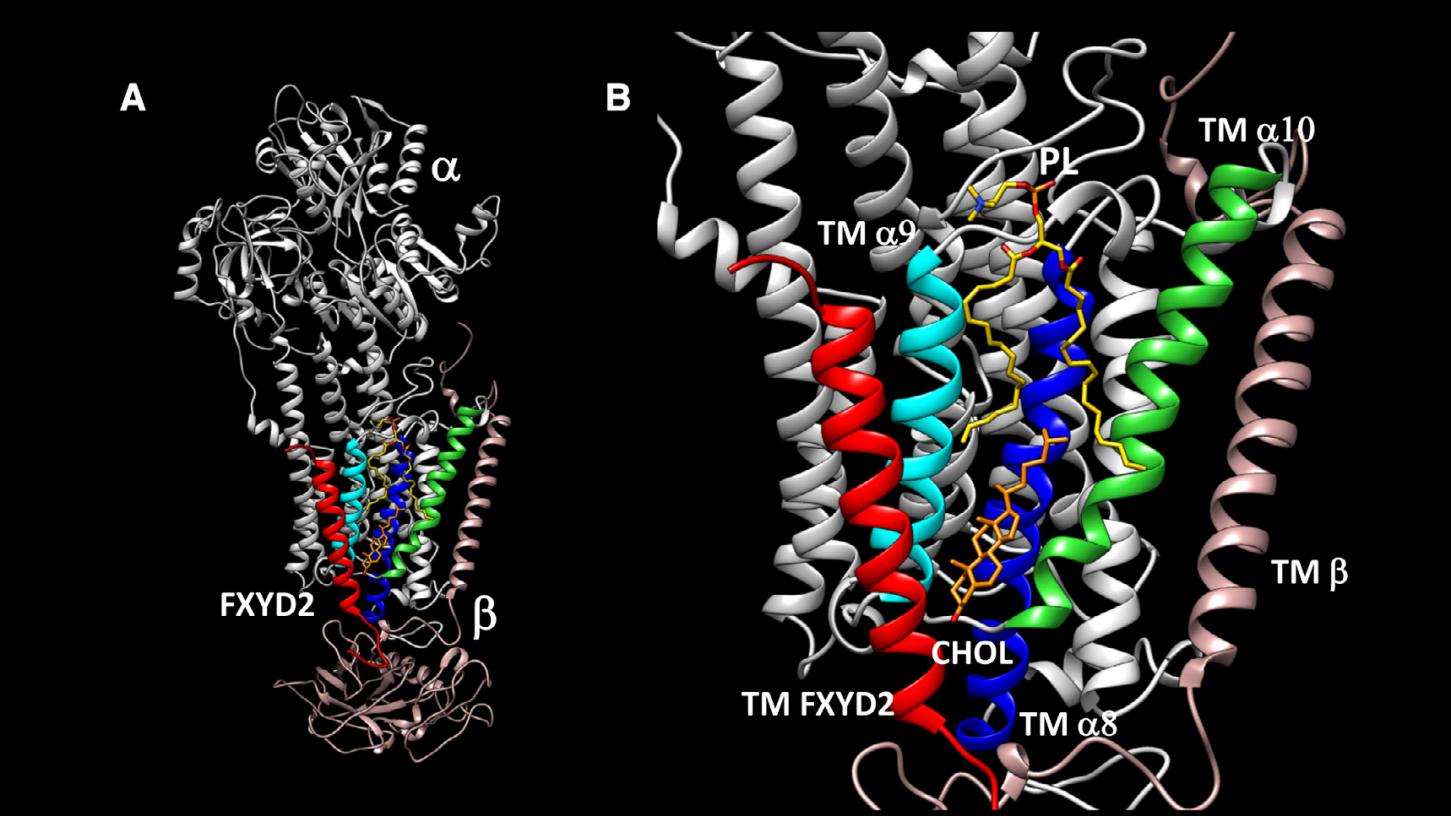
Structure of Na,K‐ATPase with specifically bound lipids 18:0‐18:1 phosphatidylserine and cholesterol. (Left) *α*,* β*, and FXYD2 subunits with bound phospholipid and cholesterol. (Right) Detail of binding site of phospholipid and cholesterol‐binding site in crevice between transmembrane segments of FXYD2, *α*M8, *α*M9 and *α*M10. Data taken from Kanai et al. (2013), Cornelius et al. (2015).

As mentioned above, renal microsomal Na,K‐ATPase in FXYD2^−/−^ mice is thermally unstable compared to wild‐type controls, showing that FXYD2 stabilizes the *αβ* subunits (Jones et al. 2005). Thermal stability has now been studied in detail using the purified human Na,K‐ATPase (*α*1*β*1 and *α*2*β*1 subunit complexes) reconstituted with purified FXYD proteins (FXYD1, FXYD2 and FXYD4) (Lifshitz et al. 2007; Mishra et al. 2011). All three FXYD proteins protect strongly against thermal (or detergent)‐mediated inactivation of Na,K‐ATPase in purified preparations and also in HeLa cells expressing FXYD1, FXYD2, and FXYD4 (Mishra et al. 2011). The molecular mechanism of stabilization has now been established. It involves a specific binding interaction between the Na,K‐ATPase and the acid phospholipid stearoyl oleoyl phosphatidyl serine (SOPS, 18:0‐18:1PS) and cholesterol within the membrane domain (Cohen et al. 2005; Haviv et al. 2007, 2013; Lifshitz et al. 2007; Kapri‐Pardes et al. 2011; Cornelius et al. 2015; Habeck et al. 2015, 2017). This is depicted in Figure 2 (in the so‐called site A ‐see (Cornelius et al. 2015)).^3^ Figure 2A provides an overview of the whole Na,K‐pump molecule including *α*1*β*1 and FXYD2 subunits with the bound lipids, while Figure 2B depicts the bound phospholipid plus cholesterol in greater detail. As shown in (Haviv et al. 2007; Kapri‐Pardes et al. 2011; Mishra et al. 2011; Habeck et al. 2015, 2017), the bound phosphatidyl serine and cholesterol interact with each other and residues in the transmembrane segments of FXYD2 and *α*M8, *α*M9 and *α*M10 to protect against thermal inactivation or excess detergent‐mediated inactivation. FXYD2 protects against thermal inactivation by amplifying the mutual *α*/FXYD2, SOPS–cholesterol interactions (Kapri‐Pardes et al. 2011; Mishra et al. 2011). Without the FXYD2 interaction, upon prolonged incubation at 37°C and higher temperatures this network of stabilizing interactions is progressively weakened and Na,K‐ATPase activity is irreversibly lost. Thermal inactivation of Na,K‐ATPase activity is associated with expulsion of *α*M8 and *α*M9 from the membrane (Arystarkhova et al. 1995; Goldshleger et al. 1995). This is critical because *α*M8 contains residues required for specific binding of Na ions (Kanai et al. 2013). The protein becomes much more susceptible to degradation by proteases. Thus, this region of the protein is a “hot‐spot” in terms of thermal destabilization and proteolytic degradation.

Based on the stabilization mechanism, we propose the following mechanism of hypermagnesuria. In kidney, over time at 37°C, the absence of *αβ*–FXYD2 interactions, will lead to destabilized and depressed Na,K‐ATPase activity and, possibly, to greater proteolytic degradation. In DCT this will compromise the ability to maintain the high intracellular K concentration that underlies the luminal membrane potential that drives Mg uptake. The end result will resemble the effect of acute Na,K‐pump inhibition by digoxin (Abu‐Amer et al. 2018). In more proximal segments (PCT and TALH) the absence of *αβ*–FXYD2 interactions should also lead to reduced Na,K‐ATPase activity and lowered Mg reabsorption but, as with digoxin, this will not be fully compensated by increased Mg uptake in DCT. The paradox of activated NCC and NKCC2 in FXYD2^−/−^ mice, without renal Na retention and hypertension (Arystarkhova et al. 2014), could then be considered, most simply, a compensatory mechanism for reduced Na reabsorption in more proximal segments (one possibility suggested in Arystarkhova et al. 2014). Note that hypocalciuria associated with the FXYD2 G41R mutation is not easily explained and reveals complexity of the mechanism.

Since FXYD2 seems to be able to act either as a negative or positive regulator of the Na,K‐ATPase one can ask how these apparently opposite effects determine the overall physiological outcome. A number of salient observations have been reported. In cultured renal or other cells, FXYD2 is not expressed, but in stress conditions, it is possible to induce expression of FXYD2, for example, by adapting cells to higher than normal tonicity (Capasso et al. 2001a, 2003a; Wetzel et al. 2004) and also to heat shock, oxidative stress and heavy metals (Wetzel et al. 2004). There is also evidence for a dynamic exchange in FXYD subunits in response to stress (e.g., FXYD2a for FXYD1) suggesting that FXYD proteins are absolutely required for cell survival in different “stress” conditions (Arystarkhova et al. 2007). In the kidney, inner medullary collecting duct (IMCD3) cells are naturally adapted to hypertonic media. In culture, IMCD3 cells grow in isotonic conditions but upon adaptation to hypertonic conditions they express greatly increased levels of *α* and *β* subunits and Na,K‐ATPase activity (Capasso et al. 2001a), as well as FXYD2b and FXYD2a (Capasso et al. 2001a). Induction of FXYD2 in the hypertonic media is essential, as seen by knock‐down of the FXYD2 by siRNA which is lethal to the IMCD3 cells in such media (Capasso et al. 2006). Interestingly, although both *α* and *β* subunits and FXYD2 are upregulated, the signaling pathways used for the subunits are different. *αβ* transcription is regulated by intracellular Na and FXYD2 by intracellular Cl, respectively (Capasso et al. 2001a, 2003b). Presumably, the strongly increased levels of *αβ* subunits, and Na/K‐ATPase activity associated with adaptation to hypertonicity (Capasso et al. 2001b) is necessary to extrude Na in the face of the increased entry of Na (and Cl). However, if expression of FXYD2 induced by the hypertonicity also raised the K_0.5_Na for activation of Na,K‐ATPase activity, as might be surmised on the basis of in vitro kinetic experiments, this would have the simultaneous effect of reducing Na/K‐pumping capacity. Hence, it is likely that stabilization by FXYD2 predominates in this physiological condition, off‐setting the possible kinetic effect of raised K_0.5_Na by preventing *α* and *β* subunit degradation and maintaining the raised Na,K‐ATPase activity required to extrude increased Na entry. In native human proximal tubule cells induction of FXYD2 by hypertonic media has also been reported to be associated with increased Na,K‐ATPase activity and *α* subunit expression (Cairo et al. 2009). The response is blunted in the cells from patients with the G41R mutation, suggestive again of the reduced FXYD2–*αβ* interaction and increased protein degradation (Cairo et al. 2009). By contrast to these results, adaptation to hypertonicity of a PCT‐derived cell line (NRK‐52E) is associated with expression of FXYD2a and also a reduction in Na,K‐ATPase activity and reduction in the rate of cell division, while knock down of the FXYD2 reverses these effects (Wetzel et al. 2004). These features are consistent with the kinetic effects of FXYD2 seen in expression experiments. The different response of these cell lines is not well understood.

Overall, it seems that the type of phenomenon that predominates may depend on the cell type, physiological setting or possibly splice variant (primarily FXYD2b in IMCD3 and FXYD2a in NRK‐52E). A simple assumption could be that in normal physiological conditions, in which the FXYD2/*αβ* expression ratio is constant, the kinetic effect of FXYD2 (increase in K_0.5_Na) dictates the response to changes in intracellular Na. In conditions in which the FXYD2–*αβ* interaction (FXYD2 R41G) or FXYD2/*αβ* expression ratio (HNF‐1B and PCBD1 mutations, FXYD2^−/−^ mice) changes, the overall cellular response is dictated primarily by destabilization or stabilization of the *αβ* complex and not by the kinetic effects. Upon reduction in FXYD2/*αβ* expression ratio or FXYD2–*αβ* interaction, destabilization and inactivation of the Na,K‐ATPase predominates. Conversely, when the FXYD2/*αβ* expression ratio increases, such as in adaptation to hypertonicity, stabilization of the *α* and *β* subunits predominates, thus reducing proteolytic degradation and maintaining increased levels of expression.

## A role for the Na,K‐ATPase in Gitelman Syndrome and Familial Hyperkalemia and Hypertension (FHHt)?

Can the rationale presented above to explain Mg handling in conditions of reduced Na,K‐ATPase–FXYD2 interactions be applied to other conditions of abnormal renal Mg handling?

### Gitelman syndrome

Gitelman syndrome is an autosomal recessive electrolyte disorder, caused by inactivating mutations in the sodium chloride co‐transporter (NCC) (Simon et al. 1996), which is expressed exclusively in DCT (McCormick and Ellison 2015). Gitelman syndrome is the most frequent inherited tubulopathy (Blanchard et al. 2017). NCC is also selectively inhibited by the thiazide diuretics used extensively to treat hypertension. Gitelman syndrome is characterized by hypokalemia and low blood pressure and hypermagnesuria with hypomagnesemia, accompanied by hypocalciuria (Blanchard et al. 2017). It is treated with dietary Mg supplements. Decreased Na absorption in DCT causes increased Na delivery to the collecting duct and a compensatory increase in Na reabsorption via epithelial Na channels (ENaC), accompanied by increased secretion of K, which causes the hypokalemia (McCormick and Ellison 2015). The hypermagnesuria has been attributed to decreased expression of TRPM6 (Nijenhuis et al. 2005), although the mechanism of cross talk between NCC mutations and the TRPM6 expression has not been elucidated (de Baaij et al. 2015a). The mechanism of hypocalciuria has been attributed to enhanced NaCl reabsorption in proximal tubule associated with ECF volume contraction (Nijenhuis et al. 2005). In humans thiazide diuretics also cause hypocalciuria and this is not associated with ECF volume contraction, leading to the suggestion that both proximal and distal tubule effects are involved (McCormick and Ellison 2015).

Overall, the mechanisms of hypermagnesuria and hypocalciuria are somewhat unclear. In this context, it is of interest that reduction in NaCl entry into DCT cells may have opposite effects on the driving forces for Mg and Ca uptake. In principle, reduced NaCl entry should lower the intracellular Na concentration and Na,K‐ATPase activity, lowering basolateral active uptake of K as well as Na efflux. The intracellular K should fall and so, also, the luminal membrane potential driving Mg entry. By contrast, at a lower intracellular Na concentration, the intracellular Ca concentration will be lowered due to coupling of Na and Ca fluxes via NCX1, and increase the driving force for luminal Ca entry. Thus, one may hypothesize that opposite effects of the NCC inhibition on driving forces for Mg and Ca entry provide a rationale of hypermagnesuria together with hypocalciuria in the DCT itself.^4^

### Familial hyperkalemia and hypertension

Familial hyperkalemia and hypertension (FHHt) also termed Pseudohypoaldosteronism type II (PHA II) or Gordon Syndrome, is the mirror image of Gitelman syndrome. FHHt is caused by mutations in the genes WNK1, WNK4, KLHL3, and Cul3 (Wilson et al. 2001; Boyden et al. 2012), which cause activation of NCC and increased sodium chloride uptake into DCT (Hadchouel et al. 2016; Shekarabi et al. 2017). The disease is inherited in an autosomal dominant way, except for recessive transmission in some cases caused by KLHL3 mutations (Boyden et al. 2012). In addition to hyperkalemia and hypertension associated with Na retention, FHHt is characterized by hypercalciuria (Mayan et al. 2002, 2015) but normomagnesuria rather than hypomagnesuria (Mayan et al. 2002). All the abnormalities are corrected by low doses of thiazides (Mayan et al. 2002). Increased Na reabsorption in the DCT reduces Na delivery to the cortical collecting duct, with lowered Na reabsorption and K secretion, thus explaining hyperkalemia. A consideration of the driving forces for Mg and Ca entry provides a rationale for hypercalciuria with normomagnesuria. Increased NCC activity should lead to increased intracellular Na and to a lowered Na gradient at the basolateral surface, and so reduced Ca extrusion on NCX1. Intracellular Ca should rise, reducing the driving force for luminal Ca entry via TRPV5, and so lead to hypercalciuria. Increased intracellular Na should increase Na,K‐ATPase activity – that is active Na extrusion and K uptake. However, because the resting intracellular K is already very high, 148 mmol/L (McCormick and Ellison 2015) any further rise must be limited by the necessity to maintain osmotic equilibrium with the extracellular fluid (310 milliosmolar), and can only be small. Thus, the membrane potential driving entry of Mg should be only slightly affected if at all.

As a final point of interest, the statement that FHHt is a mirror image of Gitelman, is reflected also in a paradoxical difference in responsiveness to thiazides. The hypokalemic, antihypertensive, and hypocalciuric effects of thiazides are much more pronounced in FHHt subjects than in controls (Mayan et al. 2002) while subjects with Gitelman have a decreased response to acute administration of thiazides (Colussi et al. 2007). Why is this paradoxical? Assuming that the rate‐limiting step for transepithelial Na transport is entry (NCC), as is usually the case in epithelia, rather than the exit (Na,K‐ATPase), one could expect Na entry via NCC to be even more rate‐limiting and thus more sensitive to thiazides in Gitelman syndrome, but less rate‐limiting and less sensitive to thiazides in FHHt. This difference has not been explained. One can speculate that thiazide binding itself might be affected differentially by changes in concentrations of intracellular Na, Cl or other ions associated with the different rates of NaCl entry (see for example (Tran et al. 1990).

## Conclusion

We have discussed several conditions in which hypermagnesuria, associated in some cases with hypomagnesemia, is attributable to inhibition or destabilization of the Na,K‐ATPase. Changes in intracellular Na are expected to change the Na,K‐ATPase activity in the DCT cells, and so affect intracellular K concentration and the driving force for transepithelial Mg transport. In this respect, Mg reabsorption in DCT may be thought of as an in vivo marker of Na,K‐ATPase activity. Based on the analysis presented here, one may anticipate that other physiological or pathophysiological conditions in which Mg handling is found to be abnormal may also reflect changes in Na,K‐ATPase activity in the DCT.

## Conflict of Interest

No conflicts of interest, financial or otherwise, are declared by the authors.

## Notes

1 The terms K_0.5_Na and K_0.5_K and KmATP refer to the concentrations of Na and K ions and concentration of ATP, respectively, required for half maximal activation of Na,K‐ATPase activity. Vmax refers to the maximal rate of Na,K‐ATPase activity at saturating concentrations of Na, K and ATP.

2 The kinetic effects of FXYD2 to reduce KmATP and K_0.5_K are unlikely to have physiologically relevance because the renal Na,K‐ATPase is largely saturated with ATP and K at normal concentrations of cytoplasmic ATP (1–2 mmol/L) and extracellular K (c. 5 mmol/L) even without the FXYD2.

3 Additional specific phospholipid–protein interactions have been identified Cornelius et al. 2015 and Habeck et al. 2015. These involve the α subunit but not the FXYD protein, and are irrelevant to the present discussion.

4 Note, for clarity, that the reduced Na,K‐ATPase activity predicted for Gitelman's syndrome is indirect, due to lowered intracellular Na, and can be contrasted with direct inhibition of Na,K‐ATPase by digoxin, which should raise intracellular Na. The opposite effects on intracellular Na should then determine the intracellular Ca concentration in DCT, leading either to an increased or decreased driving force for luminal Ca entry, in the two situations, respectively.
